# First detection of African Swine Fever Virus in *Ornithodoros porcinus *in Madagascar and new insights into tick distribution and taxonomy

**DOI:** 10.1186/1756-3305-3-115

**Published:** 2010-11-30

**Authors:** Julie Ravaomanana, Vincent Michaud, Ferran Jori, Abel Andriatsimahavandy, François Roger, Emmanuel Albina, Laurence Vial

**Affiliations:** 1 National Centre of Applicable Research for Rural Development (FOFIFA/DRZV), BP 4, Antananarivo, Madagascar; 2 Centre of International Cooperation in Agricultural Research for Development (CIRAD), CMAEE Unit, Campus International de Baillarguet, 34398 Montpellier Cedex 5, France; 3 CIRAD, AGIRs Unit, Campus International de Baillarguet, 34398 Montpellier Cedex 5, France; 4 Science Faculty, Antananarivo University, BP 566, Antananarivo, Madagascar

## Abstract

**Background:**

African Swine Fever Virus has devastated more than the half of the domestic pig population in Madagascar since its introduction, probably in 1997-1998. One of the hypotheses to explain its persistence on the island is its establishment in local *Ornithodoros *soft ticks, whose presence has been reported in the past from the north-western coast to the Central Highlands. The aim of the present study was to verify such hypothesis by conducting tick examinations in three distinct zones of pig production in Madagascar where African Swine Fever outbreaks have been regularly reported over the past decade and then to improve our knowledge on the tick distribution and taxonomy.

**Results:**

*Ornithodoros *ticks were only found in one pig farm in the village of Mahitsy, north-west of Antananarivo in the Central Highlands, whereas the tick seemed to be absent from the two other study zones near Ambatondrazaka and Marovoay. Using *16SrDNA *PCR amplification and sequencing, it was confirmed that the collected ticks belonged to the *O. porcinus *species and is closely related to the *O. p. domesticus *sub-species Walton, 1962. ASFV was detected in 7.14% (13/182) of the field ticks through the amplification of part of the viral *VP72 *gene, and their ability to maintain long-term infections was confirmed since all the ticks came from a pig building where no pigs or any other potential vertebrate hosts had been introduced for at least four years.

**Conclusions:**

Considering these results, *O. porcinus *is a reservoir for ASFV and most likely acts as vector for ASFV in Madagascar, but its apparent restricted distribution may limit its role in the epidemiology of the disease in domestic pigs.

## Background

African Swine Fever (ASF) is one of the most serious diseases in domestic pigs. It is caused by a DNA virus of the *Asfarviridae *family and usually results in acute haemorrhagic fever in susceptible animals with possibly up to 100% mortality in naïve pig herds [1]. No treatment or vaccine is currently available, and control is essentially based on preventive sanitary measures and a better knowledge of the epidemiological patterns [1,2]. ASF is enzootic in most sub-Saharan countries and its propagation is considered to be a major risk for other countries. Several introductions have been reported in the past in West Africa, Europe and the Americas, as well as more recent re-emergences of the disease in the Indian Ocean and the Caucasus [2,3].

ASF is highly contagious and is transmitted by direct contact between infected pigs and susceptible ones or by contact with infectious secretions/excretions. The virus is highly resistant in tissues and the environment, contributing to its transmission over long distances through swill feeding and fomites (e.g., contaminated material, vehicles or visitors to pig premises). In addition, African Swine Fever Virus (ASFV) can be transmitted by soft ticks of the genus *Ornithodoros*, which may either colonise pig pens in domestic areas or mammal burrows in the wild [4-6]. Considering their capacity to replicate and to maintain the virus over the years and to transmit the virus "from tick-to-tick" during mating and development stages [7-10], they are also considered excellent reservoirs of ASFV, just like wild African suids (e.g., warthogs, bush pigs and giant forest hogs). Enzootic ASF has been closely linked to the existence of *Ornithodoros *tick hosts interacting with wild suids, with *O. moubata *and *O. porcinus *ticks from the *O. moubata *species complex in East and Southern Africa [5,11], with *O. erraticus *in the Iberian Peninsula [6] and perhaps with *O. sonrai *in Senegal [12].

ASFV was probably introduced into Madagascar in 1997-1998, from the south-eastern coast of Mozambique to the south-western part of the island, with a subsequent and rapid spread to other regions [13,14]. This epizootic disease devastated more than half of the domestic pig population in Madagascar, with severe economic consequences for the local pork meat market [14]. It then evolved into an epi-enzootic pattern, with different hypothetical causes such as the adaptation of ASFV to local resistant pig populations or its establishment and persistence in native bush pigs and local *Ornithodoros *ticks, supplemented by the lack of reliable sanitary measures to control AFSV epizootic foci. The existence of bush pigs has been confirmed in forested areas of north-western, southern and eastern regions of Madagascar [15], which are considered particularly interesting interface areas for investigating the links between the ASFV sylvatic and domestic cycles. For ticks, specimens from the *O. moubata *species complex, formerly identified as *O. moubata*, were known to be present in human houses from north-western coast to Central Highlands of Madagascar and suspected of transmitting human relapsing fever [16-21]. Then, based on morphological descriptions from Walton [22], Uilenberg compared these ticks to some specimens more recently collected in pig pens and concluded that both actually corresponded to the *O. porcinus *species [23]. Finally, during the epizootic phase in 2000 following ASFV introduction, *O. porcinus *was collected in pig pens in the Antananarivo region, suggesting a possible close contact between this tick and infected pigs and a potential persistence of ASFV in *O. porcinus *tick hosts [24]. To date, no more investigations have been conducted, except the preliminary testing of 271 domestic pig sera in 2006 using an ELISA test to detect anti-*O. moubata *saliva antibodies [25,26]. This test detected 1.1% of positive sera and 39.1% of sera were considered doubtful, near Marovoay, Ambatondrazaka and Antananarivo (Ravaomanana and Vial, unpublished data).

The aims of the present work were: (i) to confirm the current presence of *O. porcinus *in pig pens in Madagascar and to better understand its geographical distribution patterns; (ii) to clarify its taxonomy and determine its phylogenetic relationships to known African *Ornithodoros *species and subspecies; and (iii) to detect natural long-term infections by ASFV in the Malagasy *O. porcinus *and to evaluate its potential role in ASFV domestic cycle of transmission in Madagascar.

## Methods

### Study zones and periods for field investigations

**Figure 1:**
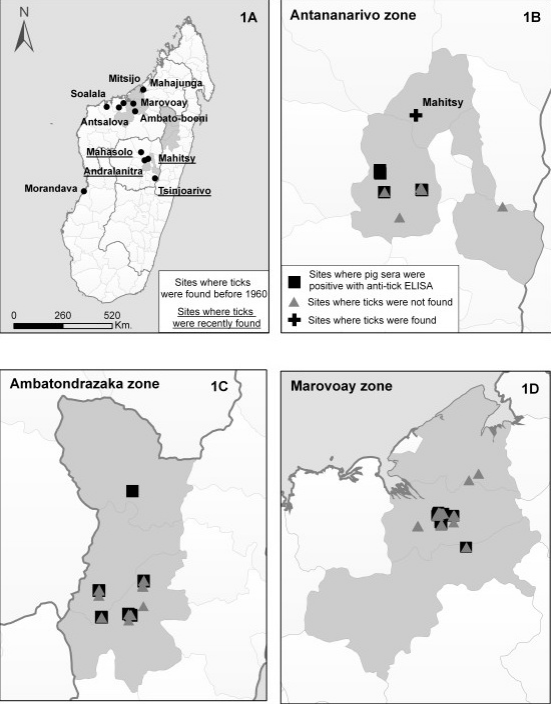
**Geographical location of the study zones in Madagascar**. (1A) presents the geographical sites where *Ornithodoros *soft ticks have been previously reported (dark dots with non-underlined names for sites where ticks were found before 1960 and underlined names for those where ticks were recently found). (1B), (1C) and (1D) present specific maps for the Antananarivo, Ambatondrazaka and Marovoay zones, respectively, pinpointing sites where pig sera were positive with the anti-tick ELISA test (dark squares), sites where tick examinations were conducted without finding ticks (grey triangles) and sites where ticks were found (dark crosses).

Three study zones (Antananarivo, Ambatondrazaka and Marovoay) (Figure 1) were selected and tick examinations in pig pens were conducted during the dry season from July to December in 2006 (Marovoay) and 2007 (Antananarivo and Ambatondrazaka). Only four additional farms were visited in 2008 in the Antananarivo zone to investigate the local distribution of *O. porcinus*. The three zones had large pig populations (from 8% in the Ambatondrazaka zone to 40% in the Antananarivo zone of the total Malagasy pig population) and ASF outbreaks had been regularly reported on farms, with an estimated incidence ranging from 15.2% to 42.5%, depending on the area (Costard, personal communications). Pig production systems varied from traditional small farms in the Marovoay and Ambatondrazaka zones to more industrialised practices for commercial purposes in the Antananarivo zone.

The Marovoay zone consists of a dry and hot tropical coastal zone, characterised by a dry climate throughout the year. The presence of *O. porcinus *(formerly *O. moubata*) has been reported on the west coast from Mahajunga to Morondava since the 18^th ^century by Drury and confirmed by many authors in the first half of the 20^th ^century [16-21]. However, more recent investigations suggested their quasi extinction since the 1950 s, at least from Soalala to Mahajunga [24,27].

The Ambatondrazaka zone consists of a wet and hot tropical coastal area. The climate is characterised by rainfall throughout the year, especially from December to March. The presence of soft ticks has never been demonstrated in this zone.

The Antananarivo zone consists of highlands characterised by a high-altitude climate, with a hot and wet season from October to February and a dry and cold season from March to September. Although altitude is usually considered unsuitable for the presence of *Ornithodoros *ticks [28], specimens of *O. porcinus *were found inside human and animal houses from the 1960 s to 1980 s in Mahasolo, 150 km from Antananarivo [23,27], and in 2000 in Mahitsy, Andralanitra and Tsinjoarivo, north-west of Antananarivo and south of Antananarivo, respectively [24].

### Tick collections in pig pens

In the Marovoay and Ambatondrazaka zones, a random cluster sampling by villages was conducted. The sample size (i.e., the number of farms to be examined in each village of the study zone) was determined using the "Detection of disease" module of the WINEPISCOPE 2.0. software (free download at http://www.clive.ed.ac.uk/winepiscope). It was based on the estimated total number of pig farms and the estimated proportion of pig farms to be detected as infested by *O. porcinus*, with a confidence interval of 95%. The villages examined for tick presence were selected from a preliminary farm census taken in 2006 in the three study zones for domestic pig sampling and were, as much as possible, homogenously distributed within each study zone. An estimated prevalence of infestation of 30% was used based on previous studies conducted on *Ornithodoros *ticks colonizing pig farms in Spain and Africa [5,6]. For the Antananarivo zone, we targeted pig farms that were known to be infested in 2000 [24]. Random sampling was then conducted in the Arivonimamo village (Figure 1). For the three study zones, one quarter of pig farms that were included in the random sampling were previously detected positive or doubtful using the anti-tick ELISA test in 2006. Finally, if the presence of *O. porcinus *was confirmed, local investigations were conducted around infested farms in order to determine local tick distribution patterns and the size of tick foci.

Ticks were manually extracted from accessible crevices, small holes and external structures of pig pens, whereas deeper fissures were examined using a portable gasoline-powered vacuum cleaner adapted for burrow-dwelling ticks [29]. The content of the collected dust was laid out in a white tray and exposed to the sun in order to collect *Ornithodoros *soft ticks that are photophobic [30]. Live ticks were placed in small plastic boxes, each of them containing a wet piece of filter paper to maintain a relative humidity suitable for tick survival. In the laboratory, ticks were stored at -80°C until analysis.

### Molecular analyses on ticks

*O. porcinus *ticks collected during the study, as well as specimens available from previous samplings in 2000, were identified according to morphological features, sex and developmental stage [22,23]. After three washings in 1% PBS, a tick homogenate was obtained for each specimen by crushing the tick in 1 ml of MEM supplemented with 50 U/ml of penicillin, streptomycin and fungizone in a 1.5-ml Eppendorf tube. Tick homogenates were frozen twice at -70°C overnight and then vortexed, to facilitate DNA accessibility before storage at -70°C.

For molecular use, tick homogenates were centrifuged and DNA extraction was carried out on 200 μL of the supernatant using Phenol Chloroform [31]. In order to test the integrity of the extracted DNA, *16 S rDNA *of each tick was amplified by polymerase chain reaction (PCR) using primers designed specifically for ticks belonging to the *O. moubata *species complex according to the PCR protocol described by Vial [32]. Positive samples were then tested for ASFV infection using a nested PCR that amplified part of the *VP72 *gene and that was described by Basto [33]. All PCR products were detected by electrophoresis on 1.5% agarose gel in TAE 1x buffer (40 mM Tris acetate, 2 mM EDTA, pH 8.3) and stained with ethidium bromide.

### Phylogenetic analyses

To determine phylogenetic relationships of collected ticks and to confirm ASFV infections, some *16 S rDNA *and *VP72 *PCR products were directly sequenced using an ABI PRISM 3730xl DNA analyser (Cogenix Meylan, France). Sequences were edited and manually aligned using Seaview software [34]. Phylogenetic trees based on molecular sequences were constructed by applying the neighbor-joining (NJ), the parsimony (P) and the maximum likelihood (ML) algorithms of the Phylip software package, using 1000 bootstraps (BS) with random sequence addition [35]. Single deletion was treated as a fifth base. *VP72 *amplified sequences were compared to the complete sequence of the gene available for the reference ASFV strain BA71V (M34142 in GenBank). For *16 S rDNA *alignment, the soft tick *Argas persicus *from the Argasinae sub-family was used as the outgroup (L34321 in GenBank). Available *16 S rDNA *sequences of African specimens from the *O. moubata *species complex were included to refine phylogenetic relationships of Malagasy ticks, especially the reference sequences for *O. moubata *sensu strict Murray, 1877 (L34328 in GenBank), *O. porcinus porcinus *Walton, 1962 (L34329 in GenBank) and *O. porcinus domesticus *Walton, 1962 (L34330 in GenBank) published by Black [36].

## Results

### Presence of *O. porcinus *in Malagasy pig pens

**Figure 2:**
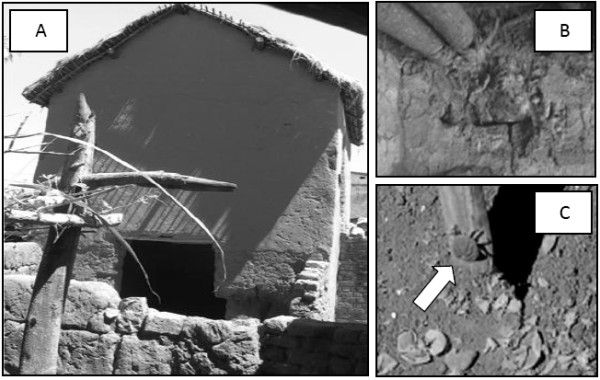
**Infested pig farm in Mahitsy (Madagascar)**. (2A) presents the building where *O. porcinus *ticks have been found. This building is typical from the Antananarivo zone with sand or mud soils, mud or concrete brick walls with few or no apertures except the door, and is located at the ground level of a two-storey human dwelling. (2B) presents one of the cracks inside the building where ticks were found and (2C) shows one tick (pointed by a white arrow) and many sloughed shins collected from these cracks.

*O. porcinus *was confirmed in one farm located in Mahitsy (18°45'3''S, 47°20'47''E) in the Antananarivo zone (Figure 1 Figure 2). Four other farms were examined in its 20-2000 m periphery without finding any other ticks (Table 1). No tick was found in the nine additional farms that were visited in the Arivonimamo village from the same zone neither among the 70 farms examined in the two other zones (Table 1). In the first zone, it was no more possible to take samples in the Andralanitra and Tsinjoarivo villages because of the recent destruction of the pig premises. In the Marovoay and Ambatondrazaka zones, sampling was incomplete due to ASF foci occurring during the study period and that prevented to finish field investigations for biosafety reasons. As a result, 40 pig pens were visited in the Marovoay zone instead of the 42 expected, with a confidence level ranging from 90% for Andranofasika to 95% for the other villages. In the Ambatondrazaka zone, 30 pig pens were accessible for tick examination instead of the 35 expected, with a confidence ranging from 92% to 95% and decreasing to 88% for Bejofo.

**Table 1:** Sampling protocol and results for tick examinations on pig farms in the three study zones of Madagascar.

Zone	Village	Aprox. no. of farms per village	Sample size for detection	Visited farms	Confidence level for detection (%)	No. of infested farms per village
Marovoay	Marovoay/Ankazomborona	300-450	9	9	95	0
	Manaratsandry	100-150	9	9	95	0
	Andranofasika	25-50	8	6	90	0
	Tsararano	50-75	8	8	95	0
	Bekobay/Tsilakanina	35-50	8	8	95	0

Ambatondrazaka	Bejofo	100-150	9	6	88	0
	Ambatosorotra	100-150	9	8	94	0
	Ambohimandroso	50-100	8	7	92	0
	Ambatondrazaka	200-250	9	9	95	0

Antananarivo	Arivonimamo	200-250	9	9	95	0
	Mahitsy	-	-	5	-	1

The table indicates the villages that have been visited, the estimated number of farms to be visited according to the estimated number of existing farms in each village and the real number of farms that have been examined for ticks. Then, the number of infested farms is presented for each village.

### Molecular identification and phylogeny of Malagasy *O. porcinus*

**Figure 3:**
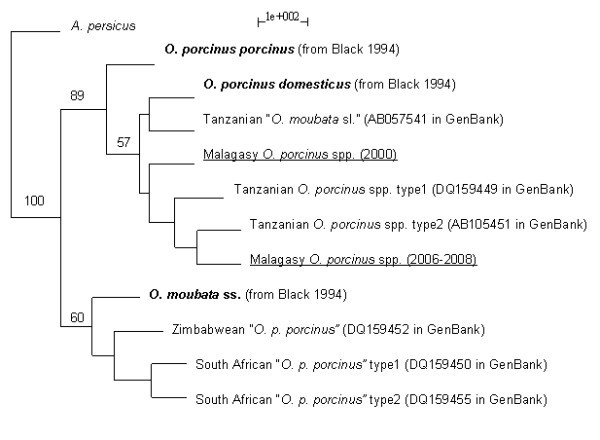
**Phylogeny of Malagasy *O. porcinus *spp. ticks**. This represents a rooted consensus phylogenetic tree of *16 S rDNA *sequences from Malagasy *O. porcinus *spp. ticks collected in pig pens in 2000 and 2006-2008 (underlined names), as well as *O. moubata *sensu stricto, *O. p. porcinus *and *O. p. domesticus *reference sequences (bold names) and some other sequences available in GenBank for the *O. moubata *complex of species, using the Maximum Likelihood (ML) method, with 1000 bootstraps and random sequence addition. Only bootstraps up to 50% have been indicated and identical sequences have been removed for simplification. Incorrect names given to specimens during field collection are indicated in quotes.

Three *16 S rDNA *sequences from ticks collected in 2000 in Mahitsy and three from the same location in 2007-2008 were produced. The amplified sequences were identical among ticks from 2000 (HM588698 in GenBank) and 2007-2008 (HM588699 in GenBank), respectively. 273 bps were aligned with available published sequences for phylogenetic analysis. Ninety-four sites were variable and 44 were parsimony informative for the whole dataset (differences at one site represented in at least two different sequences). Within the *O. moubata *species complex, 50 sites were variables and 22 were parsimony informative. Malagasy ticks from 2000 and 2006-2008 differed by only one substitution. NJ, P and ML trees had the same configuration. The consensus tree from the ML method is shown on Figure 3. The monophily of the *O. moubata *species complex and the distinction of *O. moubata *sensu stricto and *O. porcinus *were supported by 100% BS values*. O. p. porcinus *and *O. p. domesticus *were differentiated with a 89% BS support. Specimens from South Africa and Zimbabwe that were named *O. porcinus *fell into the *O. moubata *sensu stricto group with a 60% BS support. Malagasy ticks were likely related with the reference specimen of *O. p. domesticus *with a 57% BS support, and to Tanzanian ticks collected in the 1980 s and, more recently, in 2003.

### Detection of ASFV natural infection in Malagasy *O. porcinus*

Using PCR amplification of the *VP72 *virus gene, ASFV infection was detected in 13 ticks out of 182 positive ones for 16 S rDNA. All stages and sexes were found to be infected. Two out of 21 tested ticks (9.52% with 95% CI [8.94-10.09]) were positive in 2000 and 11 out of 161 (6.83% with 95% CI [6.34-7.32]) in 2007-2008. The sequencing and the alignment of 243 bps from two amplified fragments (HM588697 in GenBank) confirmed the detection of ASFV since they showed 100% homology with *VP72 *amplified from BA71V.

## Discussion

In this present study, *O. porcinus *was found on a traditional pig farm in Mahitsy in the Antanananarivo zone (Madagascar), confirming previous results related to the presence of *O. porcinus *on the island. The fact that ticks were found in the same pig pen after an interval of seven years, whereas they were absent from other neighbouring farms, suggests their high propensity to establish stable focal tick populations and their low dispersal capacity. These characteristics have been reported for several *Ornithodoros *tick species and are linked both to their endophilous lifestyle and their ability to feed rapidly on their hosts (a few minutes to one hour, at least for nymphs and adults) [28,30]. These ticks colonize nests, burrows and caves of vertebrate animals or cracks and crevices in human and livestock habitats. They reduce possible exposure to unfavourable external conditions by rapidly detaching from their vertebrate hosts before these hosts leave the habitat, and by remaining in their underground sheltered habitat. If potential hosts are absent from the habitat, these ticks are also able to survive for several years without feeding, provided the microclimate of their habitat is sufficiently suitable [30].

Our investigations also confirmed that *O. porcinus *from Madagascar can be infected by ASFV since natural infections with ASFV were detected in about 8% of tick specimens collected from the Mahitsy farm. Since 2004 until now, the owner of this farm has abandoned pig production since any pigs he has introduced in these pig buildings systematically died from ASFV. Thus the detection of infections in ticks collected in 2000 and then in 2007 from the same pig premises without any introduction of potentially infected pigs (the only confirmed susceptible vertebrate hosts for ASFV with wild suids) for at least three years suggested the capacity of long-term persistence of AFSV in Malagasy *O. porcinus*. This is in agreement with previous surveys conducted on African *Ornithodoros *tick vectors concerning virus persistence in the same individuals or among tick populations through transovarial and veneral transmissions [8,9]. For the first time, there is field evidence that *O. porcinus *in Madagascar is a natural reservoir for ASFV that most probably also acts as a competent vector. Experimental transmission studies are needed to verify these assumptions.

Furthermore, we were able to clarify the phylogenetic status of *O. porcinus *ticks that colonise pig pens in Madagascar. As previously suggested by Uilenberg [23], these ticks belong to the *O. p. domesticus *sub-species Walton, 1962, although they share some morphological and genetic characteristics of the closely related *O. p. porcinus *Walton, 1962. They are phylogenetically very close to Tanzanian soft ticks that transmit *Borrelia duttonii*, the agent responsible for human tick-borne relapsing fever in this region [37]. This result suggests that the same *O. porcinus *ticks may be able to maintain and transmit both a human pathogen and an animal pathogen by shifting vertebrate hosts, depending on their availability in their habitat. This is a typical strategy of indiscriminate host feeders to increase the amount of potential hosts and may be interpreted as an adaptation to their endophilous lifestyle [30].

Finally, the distribution range of *O. porcinus *seems to be limited to tiny heterogeneous populations in Madagascar, since ticks were only found in one village of the Central Highlands and seem to be absent from the Morovoay and Ambatondrazaka zones. If such a restricted geographical range is confirmed, the epidemiological role of *O. porcinus *in the persistence of ASFV in Malagasy domestic pigs may be low. These distribution patterns confirm past studies indicating the quasi extinction of this tick since the 1950 s from the north-western part of the country and the existence of a remaining isolated pocket in the centre of the island today [24,27]. However, they are not consistent with previous serological anti-*O. moubata *ELISA tests conducted in 2006, which suggested the presence of *Ornithodoros *ticks in the three study zones. The intrinsic specificity of this test [26,38] and its reliability for the specific Malagasy situation with the *O. porcinus *species have been questioned. Our target sample size was also not met but the maximum risk to not detect an infested farm did not exceed 12%. In addition, our sampling effort might not have revealed tick presence if the real proportion of infested pig pens was lower than the expected prevalence of 30% established as the threshold in this study. However, this threshold corresponds to the lowest margin that has been observed in endemic African countries and Spain [5,6]. Many interrelated factors may explain the restricted distribution of *O. porcinus *in Madagascar. Cool to hot temperatures from the Marovoay and Ambatondrazaka zones are suitable for the survival and the development of *O. porcinus *but the traditional open buildings typically used for pig rearing in these regions may not provide appropriate habitats for endophilous and photophobic soft ticks [28,30]. Conversely, under the harsh and apparently unfavourable climatic conditions of the Central Highlands in the Antananarivo zone, *O. porcinus *may survive and complete its development cycle in the traditional closed pig buildings encountered in this region, which maintain suitable darkness and humidity for soft ticks and buffer external climatic variations. Locally, the frequency of pig building disinfections using Cresyl (effective acaricide, in addition to its antibacterial, antiviral and antifungal properties), as mentioned by some farmers, may prevent tick establishment. Further investigations on probable recent climate changes in Madagascar and anthropogenic effects such as the increasing use of insecticides for controlling plague or malaria, or the improvement of hygiene in households should also be encouraged in order to identify other factors that might have promoted consistent and permanent modifications in the distribution patterns of *O. porcinus *in Madagascar.

## Conclusions

Our examinations of Malagasy pig farms confirmed the presence of a soft tick belonging to the *O. porcinus domesticus *sub-species Walton, 1962 on the island, at least at one site, and its natural long-term infection by ASFV. The role of this argasid tick in virus maintenance and probably transmission in endemic areas seems to be relevant. However, for unknown reasons, its distribution range in Madagascar seems to have considerably declined over these last decades. Only small scattered infested foci may remain, which might moderate the role of this soft tick in the observed persistence of ASFV in Malagasy domestic pigs since its introduction more than ten years ago.

## Competing interests

The authors declare that they have no competing interests.

## Authors' contributions

JR performed field tick collections and laboratory analyses on tick and pig samples. She wrote part of the methods. VM and EA supervised laboratory analyses. FJ, AA and FR assisted with field investigations and data analyses. LV designed the sampling protocol, supervised field investigations, analysed data and wrote the remaining sections of the paper. All authors read and approved the final manuscript.
